# A method for the geometric calibration of ultrasound transducer arrays with arbitrary geometries

**DOI:** 10.1016/j.pacs.2023.100520

**Published:** 2023-06-07

**Authors:** Karteekeya Sastry, Yang Zhang, Peng Hu, Yilin Luo, Xin Tong, Shuai Na, Lihong V. Wang

**Affiliations:** a Caltech Optical Imaging Laboratory, Department of Electrical Engineering, California Institute of Technology, 1200 East California Boulevard, Pasadena, CA 91125, USA; b Caltech Optical Imaging Laboratory, Andrew and Peggy Cherng Department of Medical Engineering, California Institute of Technology, 1200 East California Boulevard, Pasadena, CA 91125, USA

**Keywords:** Photoacoustic imaging, Ultrasound transducer arrays, Geometric calibration, Position estimation

## Abstract

Geometric calibration of ultrasound transducer arrays is critical to optimizing the performance of photoacoustic computed tomography (PACT) systems. We present a geometric calibration method that is applicable to a wide range of PACT systems. We obtain the speed of sound and point source locations using surrogate methods, which results in a linear problem in the transducer coordinates. We characterize the estimation error, which informs our choice of the point source arrangement. We demonstrate our method in a three-dimensional PACT system and show that our method improves the contrast-to-noise ratio, the size, and the spread of point source reconstructions by $\left(80\pm 19\right)\%$, $\left(19\pm 3\right)\%$, and $\left(7\pm 1\right)\%$, respectively. We reconstruct the images of a healthy human breast before and after calibration and find that the calibrated image reveals vasculatures that were previously invisible. Our work introduces a method for geometric calibration in PACT and paves the way for improving PACT image quality.

## 1 Introduction

Photoacoustic computed tomography (PACT) [1], [2], [3] is an emerging hybrid medical imaging modality that combines the molecular specificity of optical imaging and the low tissue scattering property of ultrasound to provide deep tissue imaging with optical absorption contrast. A typical PACT system consists of a laser for light delivery to the target, an ultrasound transducer array for acoustic detection, and a data acquisition system for recording and digitizing the photoacoustic (PA) signals. The ultrasound transducer arrays used in PACT come in various geometries and have different operating frequencies and bandwidths [4], [5], [6], [7], [8]. Knowledge of the exact locations of the transducers in these arrays is crucial to reconstructing the high-contrast images that PACT is known to produce. However, due to manufacturing errors, the positions of the transducers in the manufactured array do not exactly match those in the design, which degrades the reconstructed image quality. Correcting these errors is essential for maximizing the potential of PACT systems.

The problem of position estimation has been studied extensively in fields such as global positioning systems (GPS) [9], [10], wireless sensor networks [11], [12], [13], and microphone arrays [14], [15], [16]. It is typically formulated as estimating the position of an object given the times-of-arrival (ToAs) of waves (e.g., electromagnetic waves, or acoustic waves) from a few sources to the object. Generally, the positions of the sources and the wave propagation speed are assumed to be known. In the context of the geometric calibration of ultrasound transducer arrays, the major distinction is that the speed of sound in the medium is unknown. Ultrasound transducer position estimation with an unknown wave propagation speed has been studied in the context of ultrasound computed tomography [17], [18] and underwater ultrasound imaging [19], [20]. However, they also consider the element receive and transmit delays to be unknown, which leads to more involved solution strategies. In contrast, in PACT, the element receive delays can be assumed to be known since they can be found separately by diffusing laser light onto the array (which generates a strong PA signal at the instant of the light emission).

In the PACT literature, the problem of geometric calibration of transducer arrays has not been studied widely. In [21], the authors proposed a non-linear least-squares algorithm to solve for the transducer positions from ToA data collected by scanning a point source using a robotic gantry. However, they did not address the speed of sound estimation. In [8], the authors used an iterative method based on point source responses to simultaneously estimate the transducer positions, the point source positions, and the speed of sound in the medium. However, simultaneously approximating all three quantities leads to a scale ambiguity between the speed of sound and the coordinate system. Additionally, due to the non-convexity of the problem formulation, if the initial guesses of the unknowns are inaccurate, the algorithm can converge to a local minimum. Further, their method only calibrates the transducer coordinates in the radial direction and is therefore not applicable to arrays with arbitrary geometries. More recently, in [22], the authors proposed a global optimization algorithm to find the optimal location for each transducer in their 28-element transducer array by maximizing the sharpness of the reconstructed image. This method also suffers from convergence to local minima, and it scales poorly with the number of transducers. Moreover, it could lead to an unphysical situation, where different imaging targets result in different values of learned transducer coordinates. Finally, in [23], the authors circumvent the problem of geometric calibration by using deep learning-based frameworks that reduce the image artifacts resulting from the errors in the transducer positions. However, such methods suffer from a lack of interpretability [24] and result in the loss of linearity of the image reconstruction process.

In this work, we present a geometric calibration method that overcomes all the limitations stated above. We start with the point source-based formulation in [8] and reduce it to a linear system of equations in the transducer coordinates by using alternate methods to obtain the other unknown quantities in the formulation. In doing so, we overcome both the scale ambiguity between the unknowns as well as the non-convexity of the problem. Owing to the linearity of the resulting formulation, we can also derive error estimates for the estimated transducer locations. These are useful for determining the number and locations of the point sources needed to calibrate a transducer array within a given error tolerance.

The paper is structured as follows. In Section 2, we elucidate the importance of geometric calibration through numerical simulations and introduce our solution strategy. In Section 3, we apply our method to an experimental PACT system and show an improvement in the reconstructed image quality due to our method. In Section 4, we end with a discussion of our results.

## 2 Motivation and theory

Before presenting our method, we demonstrate the need for a sound geometric calibration method for PACT systems through numerical simulations.

### 2.1 Motivation for geometric calibration

**Fig. 1:**
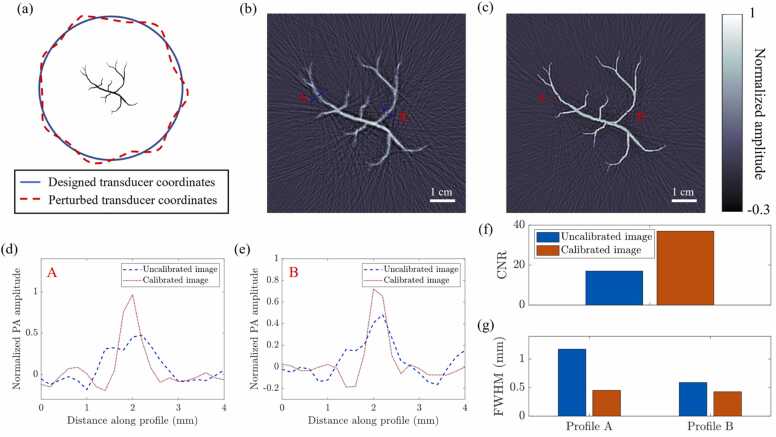
(a) Schematic of the numerical simulation to demonstrate the importance of geometric calibration (not to scale). We simulate a circular transducer array (solid blue circle) and perturb the locations of these transducers (dashed red curve). We record the PA waves at the perturbed transducer locations from an initial pressure distribution defined by a vessel-like numerical phantom placed at the center of the array. (b) Reconstructed PA image with the designed transducer coordinates (i.e., the uncalibrated image). (c) Reconstructed PA image with the perturbed transducer coordinates (i.e., the calibrated image). (d) and (e) Line profiles of the uncalibrated and calibrated images at locations A and B in the images. (f) Bar plot of the CNRs of the uncalibrated and calibrated images. (g) Bar plot of the FWHMs of the line profiles shown in (d) and (e).

Using the k-wave MATLAB package [25], we simulate a 512-element circular ultrasound transducer array with isotropic point transducers and a radius of 10 cm. Each element of the array has a Gaussian frequency response with a center frequency of 2 MHz and an ∼80 % one-way 6 dB bandwidth. Next, we perturb the x and y coordinates of each transducer with a uniformly distributed random variable in the range $\left[-0.5\lambda_{0},0.5\lambda_{0}\right]$, where $\lambda_{0}$ is the wavelength corresponding to the center frequency. This imitates the real-world situation where the actual transducer locations in an array do not exactly match the designed locations due to manufacturing errors. A schematic of this simulation setup is shown in Fig. 1(a).

We simulate the propagation of ultrasound waves due to an initial pressure distribution defined by a vessel-like numerical phantom (shown in Fig. 1(a)) and record the propagated waves at the perturbed transducer coordinates. Then, we reconstruct the images of the phantom using the designed coordinates and the perturbed coordinates, shown in Figs. 1(b) and (c), respectively. We can interpret the image obtained with the perturbed coordinates as the one obtained after geometric calibration (i.e., the calibrated image), and the image obtained with the designed coordinates as the uncalibrated one. Even for a maximum perturbation of $0.5\lambda_{0}$ (or 375μm), there is a significant degradation in the quality of the uncalibrated image (Fig. 1(b)) compared to that of the calibrated one (Fig. 1(c)) in terms of the sharpness of the reconstruction and the background artifacts.

To quantify this degradation, we compute the contrast-to-noise ratios (CNRs) of the two images in Figs. 1(b) and (c) to be 17 and 36, respectively, thus indicating a CNR reduction of as much as 50%. We also extract two line profiles from the images at locations “A” and “B” (indicated in Figs. 1(b) and (c)) and plot them in Figs. 1(d) and (e), respectively. We compute the full width at half maximum (FWHM) of each of these profiles. The FWHMs of the profiles at A and B of the uncalibrated image are 1.2 mm and 0.6 mm, whereas those of the calibrated image are 0.5 mm and 0.4 mm, respectively. We visualize the CNRs and the FWHMs as bar plots in Figs. 1(f) and (g), respectively. This simulation demonstrates the importance of geometric calibration in PACT systems.

### 2.2 Proposed method

Our method for geometric calibration is based on acquiring point source responses at various locations within the field of view (FOV) of the array. The ToA of the PA signal originating from a point source at $x\prime =[x^{\prime },y^{\prime },z^{\prime }]$ and recorded by a transducer at x=[x,y,z] can be written as,

(1) $$\frac{\mathrm{||}x-x\prime \mathrm{||}}{c}=t,$$

where t denotes the ToA of the signal, c is the speed of sound in the medium (water in this case), and ||⋅|| denotes the Euclidian norm. While the objective of geometric calibration is to estimate the transducer locations, due to the problem formulation in Eq. (1), we end up with three unknowns — the transducer location, x, the point source location, x′, and the speed of sound, c — that are related in a non-convex fashion. In addition, this problem is ill-posed because scaling both the coordinate system and the speed of sound by a constant factor will result in the same ToAs.

To overcome these issues, in our approach, we obtain the point source locations and the speed of sound in water through surrogate methods. To obtain c, we leverage the fact that the variation of the speed of sound in water with temperature has been studied extensively in the literature [26], [27], [28], [29]. We measure the water temperature accurately and infer the speed of sound from it. Next, instead of solving for the point source locations, we use a high-precision (3-axis) translation stage to move the point source to different locations within the FOV of the array. Thus, we have a coordinate system defined by the translation stage with the origin at the initial position of the stage. Having obtained the speed of sound and the point source locations, we reformulate the problem, so it becomes linear in the transducer coordinates. To do this, consider the ToA relations (Eq. (1)) for two point sources at $x_{1}^{\prime }=[x_{1}^{\prime },y_{1}^{\prime },z_{1}^{\prime }]$ and $x_{2}^{\prime }=[x_{2}^{\prime },y_{2}^{\prime },z_{2}^{\prime }]$, and a transducer at x=[x,y,z], square them and take their difference, as shown below.

$$\frac{{\left\|x-x_{1}^{\prime }\right\|}^{2}}{c^{2}}-\frac{{\left\|x-x_{2}^{\prime }\right\|}^{2}}{c^{2}}=t_{1}^{2}-t_{2}^{2}$$

(2) $$\Rightarrow \;\left(x_{2}^{\prime }-x_{1}^{\prime }\right)x+\left(y_{2}^{\prime }-y_{1}^{\prime }\right)y+\left(z_{2}^{\prime }-z_{1}^{\prime }\right)z=\frac{d_{1}^{2}-d_{2}^{2}+{r_{2}^{\prime }}^{2}-{r_{1}^{\prime }}^{2}}{2},$$

where $d_{i}=ct_{i},\;i=1,2$ are the distances between the transducer and the two point-sources, respectively, and ${r_{i}^{\prime }}^{2}=\mathrm{||}x_{i}^{\prime }|{|}^{2},\;i=1,2$. If either $r_{i}^{\prime }$ is set to zero, Eq. (2) reduces to a manifestation of the law of cosines.

**Fig. 2:**
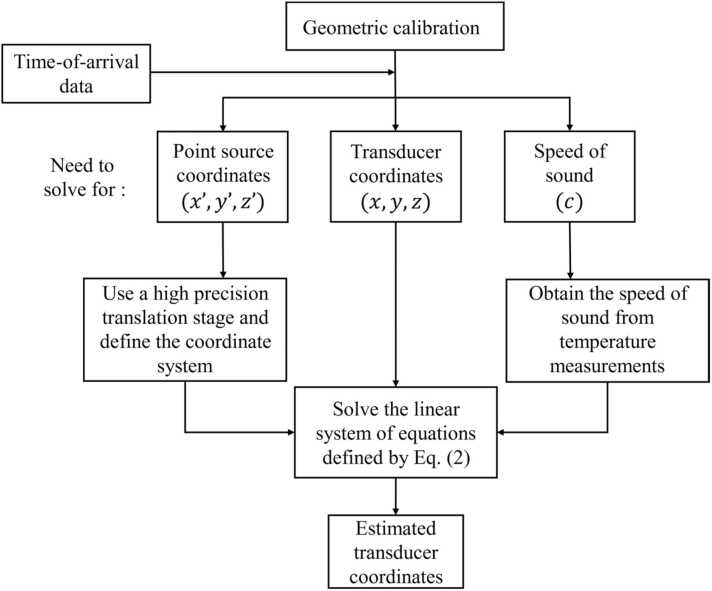
Graphical illustration of our geometric calibration method.

Now, consider the case where we have M point-source measurements. We construct Eq. (2) for each of the $N_{c}=\left(\begin{array}{c} M\\ 2 \end{array}\right)$ pairs of the point sources and solve this linear system of equations for every transducer position. Thus, the system of equations to be solved for each transducer is of the form Ax=b, where $A\in R^{N_{c}\times 3}$, $x={\left[x,y,z\right]}^{T}$ is the unknown transducer position, and $b\in R^{N_{c}\times 1}$. The $i^{\text{th}}$ row of A and the $i^{\text{th}}$ element of b have the form $a_{i}=\left[x_{\left(i,2\right)}^{\prime }-x_{\left(i,1\right)}^{\prime },y_{\left(i,2\right)}^{\prime }-y_{\left(i,1\right)}^{\prime },z_{\left(i,2\right)}^{\prime }-z_{\left(i,1\right)}^{\prime }\right]$ and $b_{i}=(d_{\left(i,1\right)}^{2}-d_{\left(i,2\right)}^{2}+r_{\left(i,2\right)}^{\prime 2}-r_{\left(i,1\right)}^{\prime 2})/2$, respectively, where (i,1) and (i,2) represent the indices corresponding to the $i^{\text{th}}$ pair of point sources out of the $N_{c}$ pairs. Finally, we estimate the transducer location using the pseudo-inverse as $\hat{x}={\left(A^{T}A\right)}^{-1}A^{T}b$. This process is repeated for each transducer independently. A graphical illustration of the proposed method is shown in Fig. 2. Posing the problem as described above allows us to characterize the error in the estimated transducer positions in a straightforward manner, as shown in Appendix A. By doing so, we can systematically choose the number and locations of point source measurements needed to calibrate the transducers within a pre-determined error tolerance.

## 3 Results

### 3.1 Methods

**Fig. 3:**
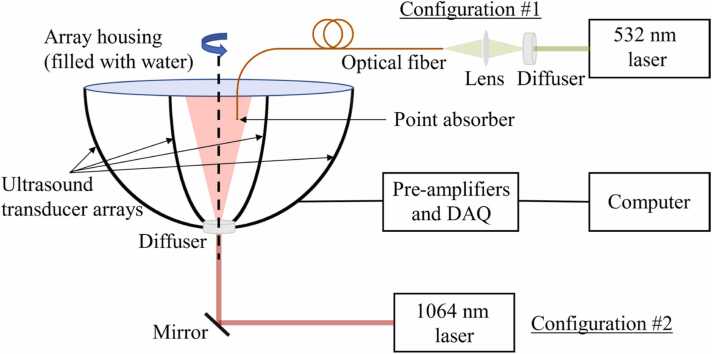
A schematic of the 3D PACT system. The system consists of four arc-shaped 256-element ultrasound transducer arrays in a hemispherical array housing, which is filled with water for acoustic coupling. The array is rotated by $90^{{}^{\circ}}$ to achieve a solid angle coverage of ∼2π steradian. The system is operated in two configurations. Configuration #1 for point source imaging: light from a 532 nm laser is coupled to an optical fiber which is terminated on the other end with an optically absorptive material, which acts as a point PA source. Configuration #2 for human breast imaging: light from a 1064 nm laser is delivered to the tissue through a diffuser (placed at the intersection of the arcs) to expand the beam.

We experimentally demonstrate our method using the 3-dimensional (3D) PACT system described in [8]. The system consists of a hemispherical array housing with four arc-shaped 256-element ultrasound transducer arrays uniformly distributed along the azimuthal direction (see Fig. 3). Each transducer element has a center frequency of 2.25 MHz and an ∼98% one-way 6 dB bandwidth. The array is rotated by $90^{{}^{\circ}}$ to achieve an ∼2π steradian solid angle coverage. The signals from each transducer are amplified and digitized by a one-to-one mapped pre-amplification and data acquisition system, and the digitized data are streamed to the computer via USB 3.0. Finally, we reconstruct the images from the raw PA signals using the universal back-projection (UBP) algorithm [30]. For the demonstration in this paper, we only consider one of the arcs.

We operate the system in two configurations. In configuration #1, meant for point source imaging, we couple 532 nm light from a laser (IS8‐2‐L, Edgewave) to an optical fiber (FG050LGA, Thorlabs; core diameter: 50μm) terminated with a light-absorbing material (carbon nanopowder), which acts as a point source for PACT. In configuration #2, meant for human breast imaging, we use a laser (LPY7875, Litron; pulse repetition frequency: 20 Hz, maximum pulse energy: ∼2.5 J) to deliver 1064 nm light to the tissue through an engineered diffuser (EDC 40, RPC Photonics Inc.) installed at the intersection of the four arcs to expand the beam. We ensure that the optical fluence on the tissue surface is within the American National Standards Institute (ANSI) safety limit at 1064 nm [31]. The two configurations are illustrated in Fig. 3.

For calibrating the array, we operated the system in configuration #1 and acquired 108 point-source responses using a high-precision 3-axis translation stage (PLS-85, Micos; bidirectional repeatability: 0.2μm) in a 6×6×3 arrangement with a pitch of 0.254 mm. We used deionized water (resistivity: 2MΩ⋅cm) in the experiment to ensure that we can accurately estimate the speed of sound. During the experiment, we measured the temperature of the water using a thermocouple (HH303, Omegaette) and inferred the speed of sound from it to be 1482.9 m/s. Note that the point source we used is not perfectly isotropic. However, this anisotropy does not affect our method as long as the signal-to-noise ratio (SNR) of the acquired data permits accurate ToA estimation.

There are several ways to estimate the ToAs of the point source signals. For instance, we can compute the noise statistics of a signal and estimate the ToA as the first instant when the signal exceeds a predefined amplitude threshold above the noise. However, the true first-arrival signal might be buried in noise, which leads to erroneous ToA estimates, especially in low SNR situations. Alternatively, we can experimentally acquire a reference PA signal with a known ToA (for e.g., by accurately measuring the distance between the source and the transducer), and use it to estimate the ToAs of the point source signals relative to the reference signal [32]. However, acquiring such a signal with a known ToA is challenging. Instead, we combine these two approaches of using noise statistics and leveraging the structure of an experimentally acquired signal. First, we find the maxima of the acquired signals, which is usually well above the noise. There is a delay between the maximum of the signal and the first-arrival due to the finite bandwidth of the transducers. To find this delay, we align the maxima of all the acquired signals and compute the average of the signals (this boosts the SNR). Then, we estimate the ToA of the averaged signal as the first instant when it exceeds a predefined amplitude threshold (three times the standard error of the noise in this case) and compute the delay between the ToA and the time when the maximum of the averaged signal occurs. Finally, we estimate the ToA of each individual signal by subtracting this delay from the time corresponding to the maximum of the signal.

We estimated the ToAs using this approach (see Supplementary Fig. 1) and applied our calibration method to estimate the locations of the transducers. The designed and calibrated locations of the transducers and their relative shifts are plotted in Supplementary Fig. 2.

### 3.2 Comparison of the images reconstructed before and after calibration

Having obtained the calibrated coordinates, we proceed to evaluate the improvement in the reconstruction quality due to the geometric calibration using two data sets. The first one consists of point source responses recorded at five different locations within the FOV of the array. Note that these data are not part of the 108 point-sources used for the calibration. The second data set is obtained by imaging the breast of a healthy adult subject lying down in a prone position within a single breath-hold of 10 seconds (to minimize motion artifacts), and with the system being operated in configuration #2.

#### 3.2.1 Point source reconstruction results

**Fig. 4:**
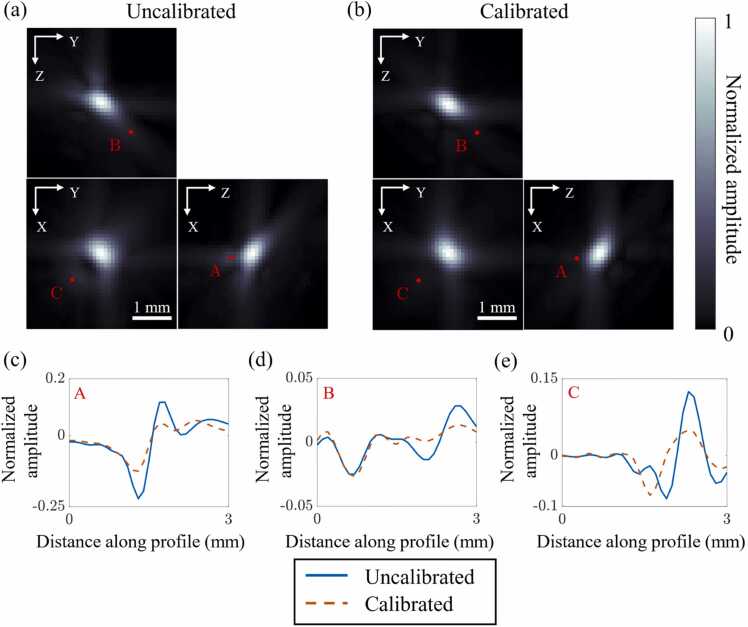
Maximum amplitude projections (MAPs) of the reconstructed volume of a point source with (a) the uncalibrated transducer coordinates and (b) the calibrated transducer coordinates. (c)-(e) Line profiles of the reconstructed volumes at points A, B, and C, respectively, in the direction perpendicular to the respective MAPs in which the points are shown.

The reconstructed images of one of the five point-sources using the uncalibrated (designed) and calibrated coordinates are shown in Figs. 4(a) and (b), respectively. The images are maximum amplitude projections (MAPs) of the reconstructed volume along the x, y, and z directions. From the images, we see a clear improvement in the calibrated image (Fig. 4(b)) compared to the uncalibrated image (Fig. 4(a)) in terms of the improved sharpness of the reconstruction and the suppressed artifacts in the background. We identify three locations in the MAPs (marked as points A, B, and C in Figs. 4(a) and (b)) where the difference between the two images is prominent and extract line profiles of the volumes at each of these locations in the direction perpendicular to their corresponding MAPs. The profiles at points A, B, and C are plotted in Figs. 4(c), (d), and (e), respectively, and they also show that the background in the calibrated image is lower than the uncalibrated one. Finally, to better appreciate the differences between the two images, we provide a video that toggles between the two images consecutively (Supplementary Video 1) and a video that shows the reconstructed 3D volumes of the uncalibrated and calibrated point sources (Supplementary Video 2).

The following is the Supplementary material related to this article Supplementary Video 1, Supplementary Video 2..

Supplementary Video 1

Supplementary Video 2

To quantify the improvement in the reconstructed point source images, we compute their CNRs. Additionally, we compute two other metrics that characterize the resolution of the system. These metrics are based on the power-RMS width (RMS stands for root-mean-square) defined in Appendix A.2 of [33]. The power-RMS width of a complex-valued function, f(t), is the standard deviation of the probability density function given by $|f\left(t\right){|}^{2}$. We extend this definition to 3D by computing the covariance matrix, $\Sigma_{V^{2}}\in R^{3\times 3}$, of the 3D distribution defined by the square of the reconstructed volume, V. From the covariance matrix, we define the following quantities,

(3) $$\text{size}\left(V\right)=\sqrt{\text{Det}(\Sigma_{V^{2}})},$$

and

(4) $$\text{spread}\left(V\right)=\sqrt{\text{Tr}(\Sigma_{V^{2}})},$$

where Det(⋅) and Tr(⋅) denote the determinant and the trace of a matrix, respectively. The size and the spread of the reconstructed point source are a measure of its volume, and its spread in the radial direction, respectively. The advantage of using these covariance matrix-based measures over conventional metrics (such as the full width at half maximum) is that they are axis-invariant and do not place assumptions on the polarity or the shape of the reconstruction (such as Gaussianity). The three metrics are computed for the reconstructions of all five point-sources and their mean and standard errors are reported in Table 1. We also report the relative improvement for each metric, defined as,

$$\begin{array}{c} \text{Relative improvement in metric}\\ =\frac{|\text{Metric of calibrated image}-\text{Metric of uncalibrated image}|\;}{\text{Metric of uncalibrated image}}. \end{array}$$

**Table 1:** A quantitative comparison of the uncalibrated and calibrated point source reconstructions using three metrics: CNR, size, and spread. The reported quantities are the mean± standard errors of the respective metrics for the reconstructed volumes of five different point sources. The size and spread of the point source reconstruction are defined in Eqs. (3) and (4), respectively.

	Uncalibrated	Calibrated	Relative improvement (%)
CNR	168±23	288±20	80±19
Size (${\text{mm}}^{3}$)	0.12±0.01	0.1±0.01	19±3
Spread (mm)	0.88±0.03	0.82±0.02	7±1

From Table 1, we see that there is an $\left(80\pm 19\right)\%$ improvement in the CNR, a $\left(19\pm 3\right)\%$ improvement in the size, and a $\left(7\pm 1\right)\%$ improvement in the spread of the point source reconstruction. We also reconstruct the image of a simulated point source (see Supplementary Fig. 3). The size and spread of the simulated point source are $0.1\;{\mathrm{mm}}^{3}$ and 0.81mm, respectively, and they are very close to the size and spread of the calibrated point source reconstruction.

#### 3.2.2 In-vivo reconstruction results

**Fig. 5:**
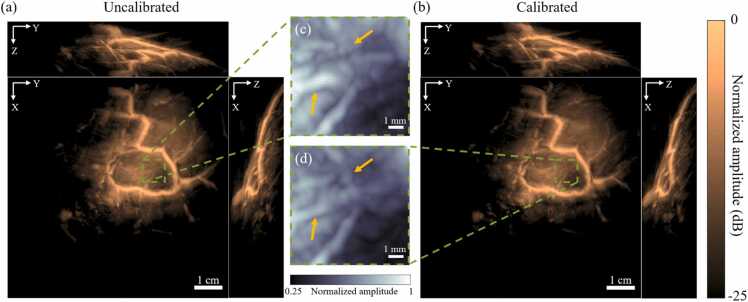
Maximum amplitude projections (MAPs) of the reconstructed volume of the breast of a healthy adult human volunteer with (a) the uncalibrated transducer coordinates and (b) the calibrated transducer coordinates. The most significant difference between these two images is observed in the region bounded by the green dashed boxes in (a) and (b), respectively. (c) and (d) show a magnified view of these regions in (a) and (b), respectively. Yellow arrows point to the X-shaped vessel structure that is visible in the calibrated image (in (d)), but not in the uncalibrated one (in (c)). Note that (c) and (d) are plotted on a linear scale.

Next, we reconstruct the images of the breast of a healthy adult volunteer with the uncalibrated and calibrated transducer coordinates and show them in Figs. 5(a) and (b), respectively. The images are the MAPs of the reconstructed volumes, and they are presented in the log scale due to the large dynamic range of the images. The major difference between the two images is in the region within the green dashed box in Figs. 5(a) and (b), where the calibrated image contains an X-shaped vessel structure that is not visible in the uncalibrated image. For better visualization, we provide a magnified view of this region for the two images in Figs. 5(c) and (d), respectively, and point to the relevant features with yellow arrows. Note that Figs. 5(c) and (d) are plotted on a linear scale. To elucidate the differences between the images in Figs. 5(c) and (d), we provide some additional visualizations. First, we provide a video that toggles between the two images consecutively (Supplementary Video 3). Next, we create a video of the reconstructed 3D volumes corresponding to Figs. 5(c) and (d) (Supplementary Video 4). Finally, we remove some of the background in the image via thresholding and show the resulting MAPs in Supplementary Fig. 4. Further, to quantify the improvement, we compute the CNRs at five points within the region of interest (the green dashed box) and compute their mean and standard error. These quantities are presented in Table 2 and they show that the CNR in this region improves by $\left(25\pm 7\right)\%$ due to the geometric calibration.

**Table 2:** A comparison of the CNRs of the images in Figs. 5(c) and (d). The reported CNRs are the mean± standard errors of the CNRs at five distinct locations in the images.

	Uncalibrated	Calibrated	Relative improvement (%)
CNR	3.9±0.6	5.0±0.9	25±7

The following is the Supplementary material related to this article Supplementary Video 3, Supplementary Video 4..

Supplementary Video 3

Supplementary Video 4

## 4 Discussion

In this paper, we have presented a method for the geometric calibration of the ultrasound transducer arrays used in PACT. The method is versatile in that it can be used for any ultrasound array, provided the point source measurements are made within the FOV of the array. The method also overcomes the ill-posedness and non-convexity of the original formulation in Eq. (1) by using surrogate methods to estimate the speed of sound and the point source locations, leading to a linear system of equations in the transducer coordinates. We applied our method to a 3D PACT system and showed that using the estimated transducer locations obtained from our method resulted in a significant improvement in the reconstructed point sources and the *in vivo* human breast image in terms of the CNR and the resolution. Our method would be particularly useful in situations where precisions in the transducer positions are difficult to control, such as when arrays are constructed using individual ultrasound transducers [7], [22].

A notable advantage of our formulation is that it is linear in the transducer coordinates. In addition to simplifying the optimization, the linearity also allows for a straightforward characterization of the error in the estimated transducer coordinates, as shown in Appendix A. Characterizing the error is particularly important for practical considerations such as choosing the number and positions of point sources needed to calibrate an array within a given error tolerance. For instance, for the demonstration in Section 3, let the error tolerance be $\lambda_{0}/5$, where $\lambda_{0}\approx 0.67$ mm is the wavelength corresponding to the center frequency of the array. For the point source arrangement that was used, as shown in Appendix A, we estimate the errors along the three coordinate axes defined by the three-axis translation stage as 0.03 mm, 0.03 mm, and 0.07 mm, respectively, which is well within our error tolerance. If our error tolerance is even lower, we can either increase the pitch between the point sources or increase the number of point source measurements to satisfy the requirement. The ability to systematically choose the point source arrangement based on an error tolerance distinguishes our method from the existing approaches in the literature [8], [21], [22].

In our method, we estimate the speed of sound in water by measuring the temperature of the water. To ensure that this estimate is accurate, a few points must be considered. Firstly, the speed of sound in water does not just depend on the temperature of the water but also its purity [34]. In our experiment, we used deionized water with a resistivity^2^ of 2MΩ⋅cm to ensure that our speed of sound estimate is accurate. Secondly, since we assume that the speed is homogeneous, we must make sure that the water temperature is uniform and constant throughout the experiment. One way to do this is to start the experiment only after the water temperature has reached a steady state as monitored at several locations and at regular intervals in time.

Our method also requires an accurate estimate of the ToAs of the point source signals. While estimating the ToAs, it is crucial to account for any delays in the data acquisition pipeline such as the element receive delays. In PACT systems, these delays can be found by diffusing the laser light onto the array which generates a strong PA signal (termed the transducer surface signal) at the instant of laser emission. The ToA estimation approach from Section 3.1 can be used to estimate the first-arrival time of the transducer surface signal. This synchronizes the laser emission with the data acquisition system. It is important to note that the ToA estimation approaches described in Section 3.1 are valid only when the point source response does not change significantly within the measurement region. If it does, then the spatial impulse response of the transducers [37] has to be incorporated into these approaches for accurate ToA estimation.

We concede that despite accounting for several factors in the estimation of the speed of sound and the ToAs, there may still be some error in these estimates. For instance, changes in the temperature that are smaller than the measurement resolution of $0.1^{{}^{\circ}}$ C could result in some error in the estimated speed of sound. Similarly, since we define our ToA based on an amplitude threshold, we ignore the part of the point source response that occurs prior to this instant, which introduces some error in our ToA estimates. We account for such errors in our error analysis in Appendix A, where we assume that our speed of sound error is 0.3 m/s (based on the resolution of the temperature measurement) and the ToA estimation error is approximately 0.45μs (based on the center frequency of the array).

While our method is readily applicable to any ultrasound transducer array, a practical concern arises when working with 2-dimensional (2D) PACT systems (for example, a ring array, or a linear array). In this case, it is crucial to ensure that the point sources and the transducer array lie in the same plane. Otherwise, the ToAs of the point source responses acquired by the PACT system do not accurately reflect the true distances between the point sources, leading to erroneous results. To overcome this, if we perform 3D geometric calibration for a 2D array, then it is necessary to account for the changes in the spatial impulse response of the transducer and the decrease in the signal-to-noise ratio while estimating the ToAs for out-of-plane point sources.

In conclusion, we presented a method for the geometric calibration of the ultrasound transducer arrays used in PACT systems, demonstrated the method in a 3D PACT system, and discussed several practical considerations in implementing the method. We hope that our work will standardize the practice of geometric calibration in PACT and lead to improved image quality in PACT systems. A demo code for our method has been posted on GitHub.^3^

## Imaging protocols

All human imaging experiments were performed with the relevant guidelines and regulations approved by the Institutional Review Board of the California Institute of Technology (Caltech). The human experiments were performed in a dedicated imaging room. Written informed consent was obtained from all the participants according to the study protocols.

Funding

This work was sponsored by the United States National Institutes of Health (NIH) grants R01 NS102213, U01NS099717, U01 EB029823, R35 CA220436 (Outstanding Investigator Award), and R01EB028277.

## Data and code availability

The data that support the findings of this study are provided within the paper and its Supplementary materials. A demo code for the calibration method has been posted online at https://github.com/karteekdhara98/PACT-geometric-calibration. The reconstruction algorithm and data processing methods can be found in the paper. The reconstruction code is not publicly available because it is proprietary and is used in licensed technologies.

## Declaration of Competing interest

L.V.W. has a financial interest in Microphotoacoustics, Inc., CalPACT, LLC, and Union Photoacoustic Technologies, Ltd., which, however, did not support this work. The other authors declare no competing interests.

## Data Availability

The reconstruction algorithm and the data that support the findings of this study are provided within the paper and its Supplementary materials. A demo code for our method has been posted online.

## Appendix A Error analysis

As described in Section 2.2, in our method, the transducer locations are estimated as $\hat{x}={\left(A^{T}A\right)}^{-1}A^{T}b$. To compute the error in $\hat{x}$, we treat the elements of b as random variables whose mean is equal to their true value and standard deviation is equal to the experimental error in their measurement. Assuming that the high-precision translation stage has an accuracy much smaller than the wavelength of the transducers being calibrated, we can treat the matrix A as a deterministic quantity with negligible error. Let the covariance matrices of b and $\hat{x}$ be $\Sigma_{b}\in R^{N_{c}{\times N}_{c}}$ and $\Sigma_{\hat{x}}\in R^{3\times 3}$, respectively. Then, we have the relation,

(5) $$\Sigma_{\hat{x}}=A^{†}\Sigma_{b}{\left(A^{†}\right)}^{T},\;$$

where $A^{†}={\left(A^{T}A\right)}^{-1}A^{T}$ is the pseudoinverse of A, and $\Sigma_{b}$ has the following form:

(6) $${\left[\Sigma_{b}\right]}_{i,j}=\{\begin{array}{cc} 0, & i\neq j;\;\left(i,1\right)\neq \left(j,1\right),\left(j,2\right);\;\left(i,2\right)\neq \left(j,1\right),\left(j,2\right)\\ \sigma_{0}^{2}d_{\left(i,1\right)}^{4}, & i\neq j;\;\left(i,1\right)=\left(j,1\right)\\ {-\sigma }_{0}^{2}d_{\left(i,1\right)}^{4}, & i\neq j;\;\left(i,1\right)=\left(j,2\right)\\ {-\sigma }_{0}^{2}d_{\left(i,2\right)}^{4}, & i\neq j;\;\left(i,2\right)=\left(j,1\right)\\ \sigma_{0}^{2}d_{\left(i,2\right)}^{4}, & i\neq j;\;\left(i,2\right)=\left(j,2\right)\\ \sigma_{0}^{2}{(d}_{\left(i,1\right)}^{4}+d_{\left(i,2\right)}^{4}), & i=j \end{array}$$

where ${\left[\Sigma_{b}\right]}_{i,j}$ is the ${\left(i,j\right)}^{\text{th}}$ element of the matrix $\Sigma_{b}$, $\left\{\left(i,1\right),\left(i,2\right)\right\}$ and $\left\{\left(j,1\right),\left(j,2\right)\right\}$ are the indices corresponding to the $i^{\text{th}}$ and $j^{\text{th}}$ combinations of point sources out of the $N_{c}$ combinations, respectively, and

(7) $$\sigma_{0}=\sqrt{{\left(\frac{\sigma_{c}}{c}\right)}^{2}+{\left(\frac{\sigma_{t}}{t_{0}}\right)}^{2}}$$

Here, $\sigma_{c}$ and $\sigma_{t}$ are the uncertainties in the measurements of the speed of sound and the ToAs, respectively, c is the speed of sound, and $t_{0}$ is the ToA of a point source response at the transducer. Since the speed of sound is derived from temperature measurements, $\sigma_{c}$ depends on the error in the temperature measurement, T. Assuming that the temperature measurement is unbiased and has an uncertainty of $\sigma_{T}$, we have $\sigma_{c}={\left|\frac{\mathrm{dc}}{\mathrm{dT}}\right|\sigma }_{T}$. The uncertainty in the ToA, $\sigma_{t}$, can be estimated as $1/f_{0}$, where $f_{0}$ is the center frequency of the array, because the bandwidth is usually given as a fraction of the center frequency. Finally, note that although the ToA from every point source is different, for simplicity, we only consider a representative ToA, $t_{0}$, for calculating $\sigma_{0}$. Having calculated $\Sigma_{b}$ using (6), (7), we can use Eq. (5) to compute $\Sigma_{\hat{x}}$. The square roots of the diagonal elements of $\Sigma_{\hat{x}}$ give us the estimation error in the transducer positions along the three coordinate axes.

To illustrate how these quantities are computed in a practical situation, we estimate the error for the demonstration in Section 3. Note that this calculation is typically performed before the experiment. Firstly, the bidirectional repeatability of our translation stage (PLS-85, Micos) is 0.2μm, which is much less than the wavelength corresponding to the center frequency of the array (670μm). Therefore, matrix A can be treated as a deterministic quantity. Assuming that the water temperature is $20^{{}^{\circ}}$ C, we infer the speed of sound to be, c=1482.3 m/s, and $\frac{\mathrm{dc}}{\mathrm{dT}}\approx 3$ m/s. Since the readings from our thermocouple (HH303, Omegaette) are sufficiently precise, we determine that the maximum error in our temperature measurement is equal to the resolution of the thermocouple, i.e., $\sigma_{T}=0.1^{{}^{\circ}}$ C. Thus, $\sigma_{c}\approx 0.3$ m/s. The center frequency of the array is 2.25 MHz. Therefore, $\sigma_{t}=\frac{1}{2.25\times 10^{6}}\approx 4.5\times 10^{-7}$ s. $t_{0}$ is estimated as the time taken for the acoustic wave to travel a distance of 13 cm (from the center of the array to the transducer), i.e., $t_{0}=\frac{0.13}{1482.3}\approx 8.8\times 10^{-5}$ s. Substituting these values in Eq. (7), we get $\sigma_{0}\approx 5\times 10^{-3}$. Next, we construct $\Sigma_{b}$ for the point source arrangement in Section 3 using Eq. (6). Then, we substitute it into Eq. (5) to obtain the errors in the estimated transducer locations along the three coordinate axes as 0.03 mm, 0.03 mm, and 0.07 mm, respectively. We study the dependence of the estimation error on the number of point sources and the point source locations in Supplementary Fig. 5.

## Footnotes

2 For reference, ultrapure water and sea water have resistivities of 18.2MΩ⋅cm[35] and 2mΩ⋅cm[36], respectively.

3 https://github.com/karteekdhara98/PACT-geometric-calibration
