# Supplementary material for: A method for the geometric calibration of ultrasound transducer arrays with arbitrary geometries
Source: Photoacoustics. 2023 Jun 7;32:100520. doi: 10.1016/j.pacs.2023.100520 (PMC10329181; doi:10.1016/j.pacs.2023.100520)
Supplement: Supplementary file 5 — Supplementary material [file mmc1.docx]

**Supplementary figures**

| 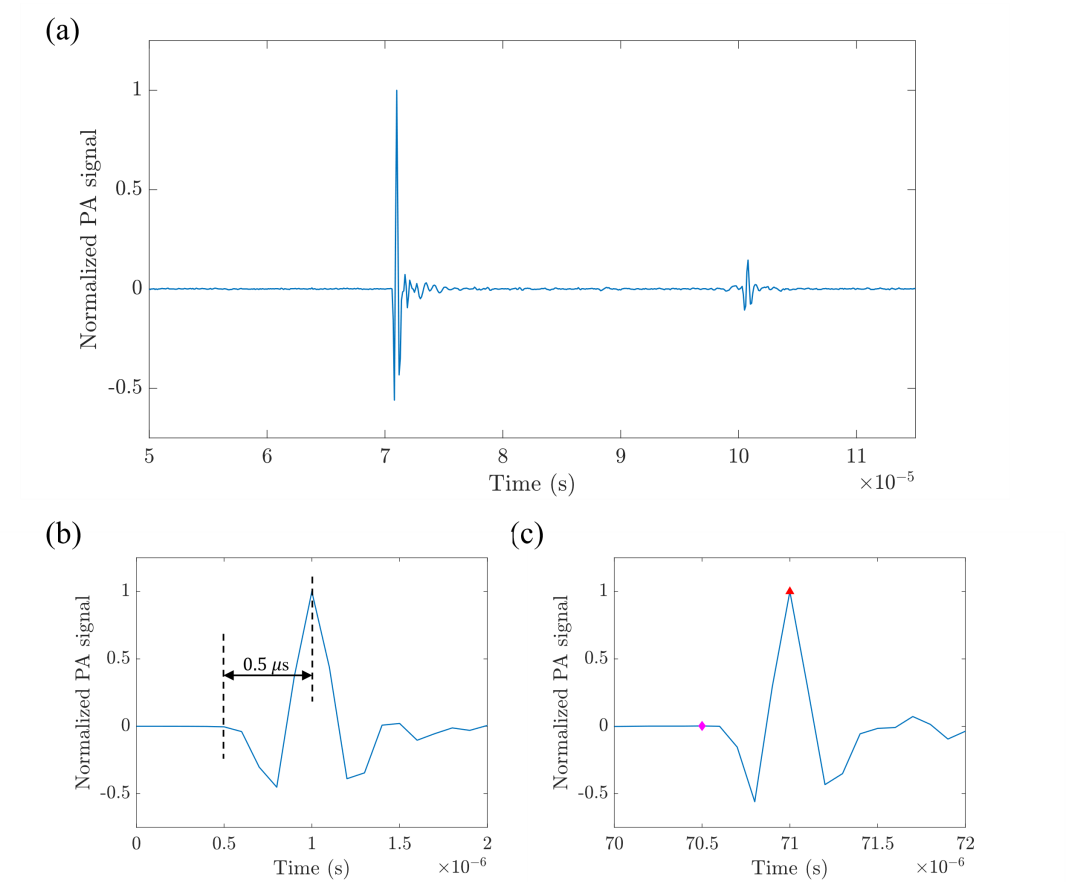 |
| --- |
| **Supplementary Fig. 1:** (a) Plot of an experimental point source response sampled at $10$MHz. The acquired signal has low noise (signal-to-noise ratio$>100$) and is composed of two distinct pulses — the earlier direct transmission signal and the subsequent weaker signal due to reflection from the water surface. (b) Plot of the averaged point source response obtained by aligning the maximums of all the acquired point source responses. The standard deviation of the noise in the averaged (and normalized) signal is $3\times{10}^{-4}$. The first arrival is determined to be at $0.5 \mu\text{s}$, and it has a normalized signal amplitude of $4\times{10}^{-3}$. Since the maximum occurs at $1 \mu\text{s}$, the delay between the maximum and the first-arrival is $0.5 \mu\text{s}$. (c) Illustration of the ToA estimation method (for the signal in (a)). We find the maximum of the signal (indicated with a red triangle) and correct for the $0.5 \mu s$ delay that occurs due to the finite bandwidth of the transducer to estimate the ToA (indicated with a magenta diamond). |

| 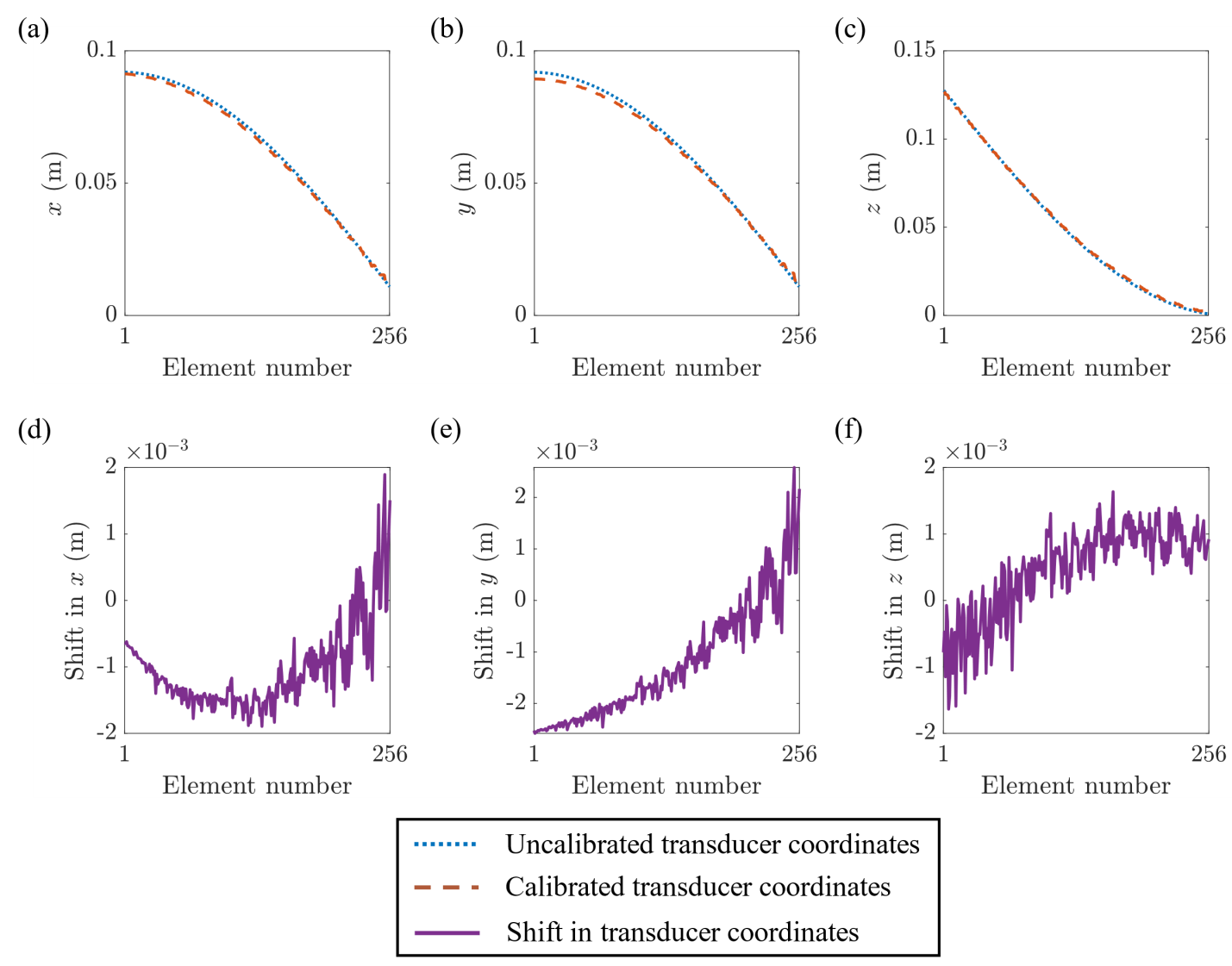 |
| --- |
| **Supplementary Fig. 2:** (a) $x$, (b) $y$, and (c) $z$ coordinates of the transducers of the $256$-element arc-shaped array introduced in Section 3.1 before and after the geometric calibration. We also plot the difference between the calibrated and uncalibrated transducer coordinates (shift in transducer coordinates) along the (d) $x$, (e) $y$, and (f) $z$ directions. The maximum shifts along the $x$, $y$, and $z$ directions are $1.9$ mm, $2.6$ mm, and $1.6$ mm, respectively. |

| 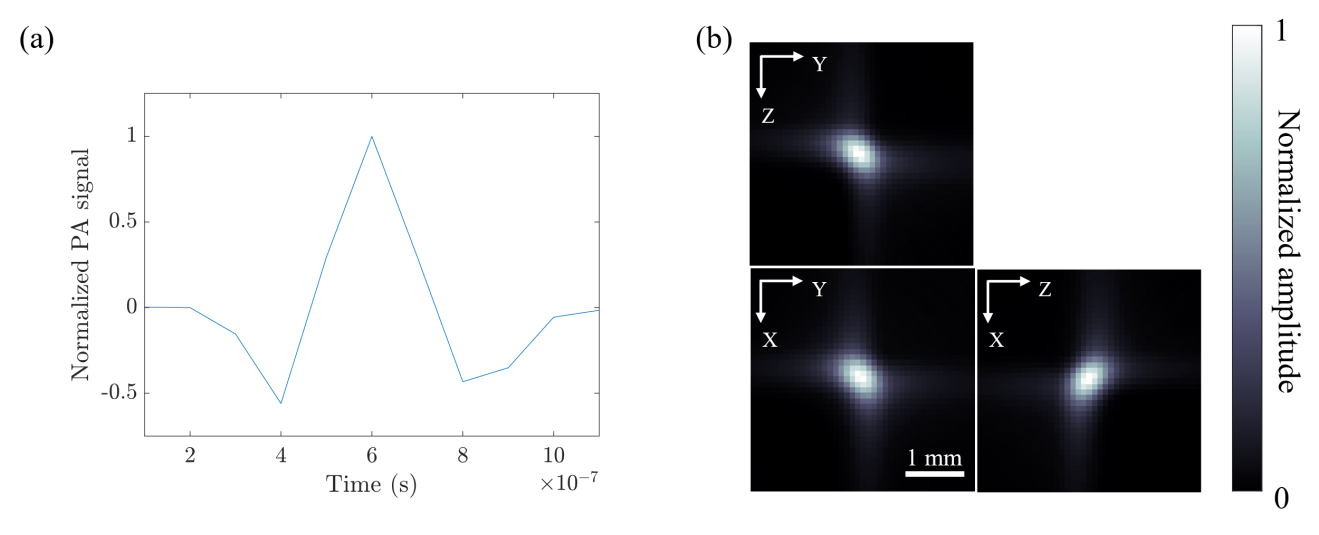 |
| --- |
| **Supplementary Fig. 3:** Image reconstruction of a simulated point source. Since running a full-scale 3D simulation in k-wave at the required discretization is computationally infeasible, we have come up with an alternate approach to perform the simulation. First, we extract a point source response from the experimental point source data. Then, we calculate the ToAs from a point source at the center of the array to the transducer locations. We shift the point source response to the appropriate arrival time for each transducer to generate the simulated point source data and reconstruct an image from this data. (a) Extracted experimental point source response (sampled at $10$ MHz). (b) Maximum amplitude projections (MAPs) of the reconstructed volume of the simulated point source. |

| 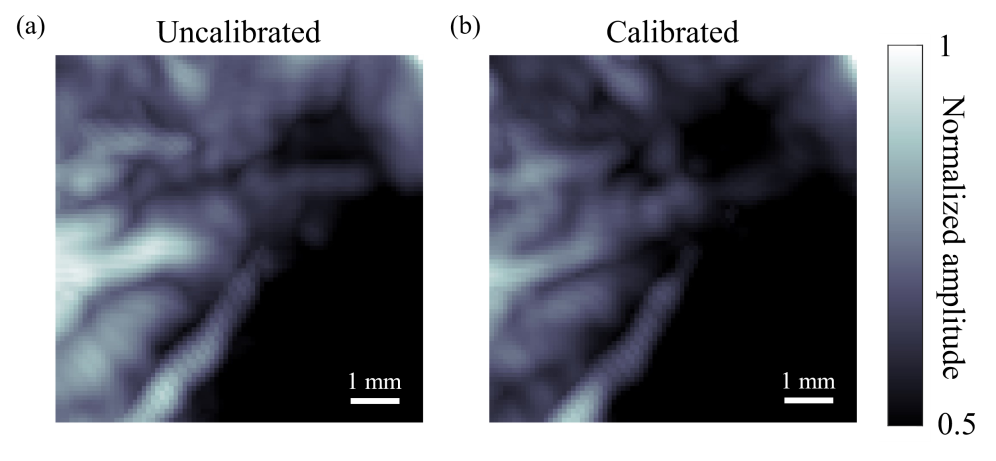 |
| --- |
| **Supplementary Fig. 4:** Visualization of the (a) uncalibrated and (b) calibrated in-vivo images shown in Figs. 5(c) and 5(d), respectively, with the normalized image pixel values thresholded between $0.5$ and $1$. This threshold reduces the background in the image and helps visualize the improvement in the image due to the calibration. |

| 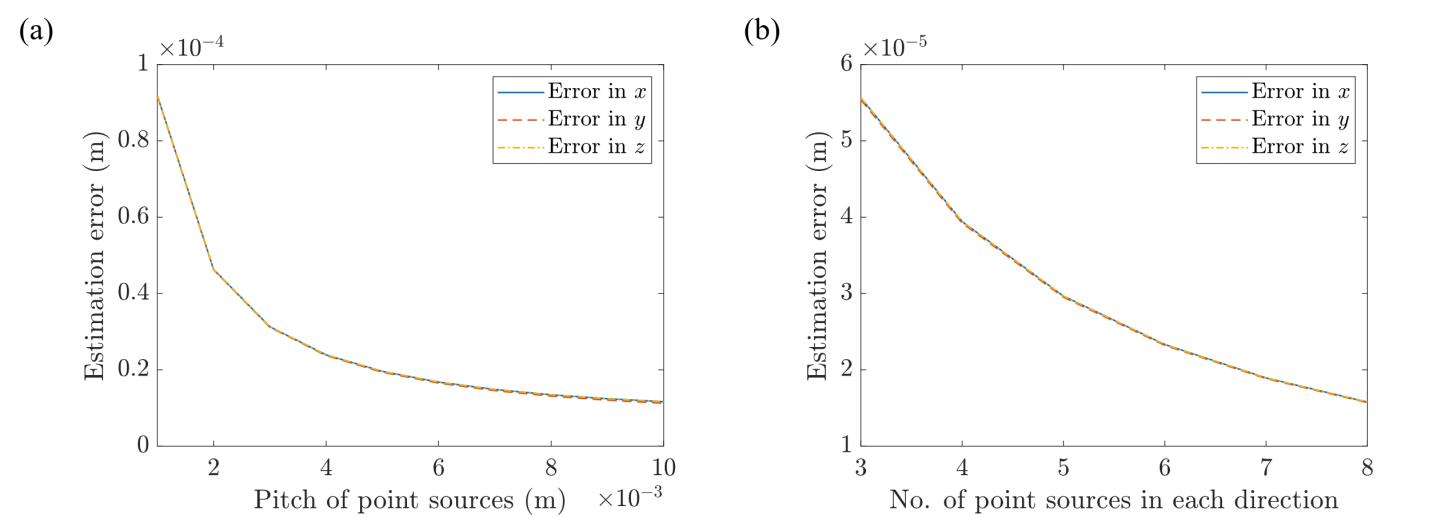 |
| --- |
| **Supplementary Fig. 5:** (a) Plot of the estimation error for a fixed number of point sources with a varying pitch. We fix the number of point sources as $125$ ($5$ in each dimension). Then, we increase the pitch of the point sources from $1$ mm to $10$ mm in increments of $1$ mm. We calculate the estimation error for each case. (b) Plot of the estimation error for a varying number of point sources within a fixed measurement region. We fix the point sources to be within a cube of side $10$ mm and discretize this volume uniformly along all three dimensions. We vary the number of point source measurements made in each dimension from $3$ to $8$ (corresponding to a total number of point source measurements ranging from $27$ to $512$). We calculate the estimation error for each case. |
